# Acknowledgement to Reviewers of *Healthcare* in 2015

**DOI:** 10.3390/healthcare4010012

**Published:** 2016-01-22

**Authors:** Healthcare Editorial Office

**Affiliations:** MDPI AG, Klybeckstrasse 64, CH-4057 Basel, Switzerland; healthcare@mdpi.com

The editors of *Healthcare* would like to express their sincere gratitude to the following reviewers for assessing manuscripts in 2015.

We greatly appreciate the contribution of expert reviewers, which is crucial to the journal’s editorial decision-making process. Several steps have been taken in 2015 to thank and acknowledge reviewers. Good, timely reviews are rewarded with a discount off their next MDPI publication. By creating an account on the submission system, reviewers can access details of their past reviews, see the comments of other reviewers, and download a letter of acknowledgement for their records. In addition, MDPI has launched a collaboration with Publons, a website that seeks to publicly acknowledge reviewers on a per journal basis. This is all done, of course, within the constraints of reviewer confidentiality. Feedback from reviewers shows that most see their task as a voluntary and mostly unseen work in service to the scientific community. We are grateful to our reviewers for the contribution they make.

Adams, James B.
Adogwa, Owoicho
Agarwal, Saurabh
Agodi, Antonella
Alves, Elisabete
Andrade, Francisco J.
Annunziato, Rachel A.
Anthony, Denis
Armstrong, Pat
Asante, A.D.
Assasi, Nazila
Astrup, Birgitte Schmidt
Austrom, Mary Guerriero
Aydede, Sema K
Bailey, Chip
Ball, Lauren
Barlow-Stewart, Kristine
Barnard, Neal
Barnes, Emily R.
Beerthuizen, Annemerle
Bell, Anthony
Belo, Isabel
Bergstrom, Nancy
Berliner, Janice L.
Bermejo, Justo Lorenzo
Bhattacharya, Kaustuv
Bingham, Daniel
Bloomfield, Hanna E.
Bloxham, Saul
Bobes-Bascaran, M.T.
Boccia, Stefania
Bogie, Kath M.
Bootsma, Martin
Borkow, Gadi
Boscart, Veronique
Boss, Lisa
Bove, Geoffrey
Bower, Peter
Bradshaw, Tim
Brennan, Frank
Bridges, Diane R.
Brienza, David
Broom, Lain
Brussoni, Mariana
Burton, Hilary
Bush, Ruth L.
Buysse, Heidi E.C.
Caracciolo, Barbara
Cárdenas, Washington B.
Carmichael, Amtul Razzaq
Caspar, Sienna
Castro-Sánchez, A.M.
Chao, Yann-Fen C.
Chew, Song Foh
Chiavaroli, Valentina
Cho, Joon Yong
Chopra, Ishveen
Cicolini, Giancarlo
Clifton, Peter
Cobelli, Claudio
Cohen, Stephanie A.
Connolly, Mark P
Cooper, Kay
Corish, Clare
Corry, Dagmar A.S.
Cousens, Nicole
Cowdell, Fiona
Cox, Diane
Coyne, Elisabeth
Crank, Helen
Crotty, Bradley H.
Cryan, John
Curran, Sarah
Damen, Saskia
Das, Rina
Davis, Claudia M.
Dawkins, Hugh J S
Day, Andrew
De la Monte, Suzanne
Dekker, Marloes
Demark-Wahnefried, Wendy
Demarré, Liesbet
Denis, Gerald V.
Derman, Peter
Dickinson, Miriam
Dreischulte, Tobias
Dunning, Trisha
Durst, Andrea
Dusso, Adriana
Dyer, Naomi
Dyjur, Louise
Ellard, David
Engelman, Alina
Erickson, Mary Ann
Escoffery, Cam
Faraci, Frank M.
Feng, Yan
Ferguson, Caleb
Foote, Celine
Forman, Daniel E.
Fortune, Darla
Francino, Pilar
Fujii, Yasuhito
Gal, Tuvia Ben
Gall, Henning
García-Sáez, Gema
Garfield, Sara
Gethin, Georgina
Gobe, Glenda C
González, Víctor M. Víctor
Good, Phillip
Gowing, Linda
Granbom, Marianne
Grant, Wiliam
Grant, Richard W.
Guallar, Pilar
Haglind, E.
Hain, Debra
Hajnsek, Martin
Halek, Margareta
Halkitis, Perry N.
Han, Kyu Yeon
Harder, Helena
Hashimoto, Ryuichiro
Hays, Ron
Heath, Gemma
Heim, Noor
Heindel, Jerrold
Heinen, Maud
Hersh, Marion
Heuberger, Roschelle A.
HIrst, Jane
Hoerr, Robert
Hogan, Anthony
Hogg, James T.
Holbrook, Joanna D.
Holding, David R.
Holley, Jean L.
Holt, Tim A
Houben, C.H.
Huikuri, Heikki
Hvalvik, Sigrun
Iizaka, Shinji
Isenberg, Sarina
Jacobs, David R.
Jaul, Ephraim
Jolley, David
Jones, Heidi E.
Joyce, Lyle D.
Juarez , Paul D.
Kardas, Przemyslaw
Katsanis, Sara Huston
Katzman, Wendy
Kaye, Elizabeth Krall
Keller, Heather
Kemp-Harper, Barbara K.
Kennerly, Susan M
Kim, Cheol-Ho
Kingsley, J. Derek
Kirchhoff, Karin T.
Kleim, Birgit
Knibb, Rebecca
Knight, Sarah
Koblin, Beryl
Kodama, Hideya
Koppel, Ross
Kornfeld, Donald
Kroeker, Karen I
Kuhn, Jens
Kullman, Emily S
Kumar, Dhavendra
Kuziemsky, Craig
Lambert, Veronica
Lantz, Ann
Latter, Susan
Laville, Maurice
Lawn, Sharon
Layman, Donald
Lee, Yau-Jiunn
Légaré, Jean-Francois
Liepelt-Scarfone , Inga
Lima, Julie C.
Lindor, Noralane M.
Lio, Antonio
Liu, Liang Qin
Lloyd, Jenny
Loeber, J. Gerard
Lohela-Karlsson, Malin
Lowres, Nicole
Luna, Aurelio
Ma, Yongjie
MacFabe, Derrick
MacLean, Michael
Maher, Chris
Malara, Alba
Malmedal, Wenche
Manninger-Wünscher, M.
Manortey, Stephen
Manzano, F.
Markofski, Melissa M.
Martinez, Homero
Martinez-Castelao, Alberto
Masella, Roberta
Mattsson, Janet
Mayhan, William G.
McClaren, Belinda J.
McGee, Jocelyn
McGrady, Meghan E.
McKenna, Malachi J
Meeker, Daniella
Melendez, Andre
Miller, Anthony B
Mitchell, Christine
Modell, Stephen M.
Moltó, José
Muraro, Antonella
Murnaghan, Donna
Murphy, Ronan
Murrock, Carolyn
Naeije, R.
Narod, Steven
Naser-ud-Din, Shazia
Neil-Sztramko, Sarah
Neymotin, Florence
Nicholls, Daniel
Nygard, Clas-Håkan
Nykänen, Irma
O'Connor, Tom
O’Reilly, Ciara
Okoroh, Juliet S.
Olver, Ian
Ooi, Keith
Oviedo-Joekes, Eugenia
Pace-Schott, Edward F.
Pal, Tuya
Palermo, Claire
Pan, Eric C.
PAN, Xin
Parker, Christina
Patarrão, Rita
Pauline, Sung Chan Po-lin
Peipert, Jeffrey F
Perry, Tam E.
Peterson, Michael
Phillips, Siobhan M
Pichon, Latrice C.
Pieper, Barbara
Pit, Sabrina W.
Polk, Donna M.
Pollitt, Rodney J.
Prescott, Gina M.
Price, Patricia
Probst, Sebastian
Quigley, Denise D.
Quintas, Rui
Raghupathi, Wullianallur
Rahman, Momotazur
Ramirez, Cherie L.
Ray, Udayan
Ray, Daniel E.
Reed, William R.
Reeve, Joanne
Reid, Joanne
Requena, Teresa
Ribeiro, Rogerio
Rinaudo, Paolo
Roberts, Shelley
Ross, Alastair B.
Rossignol, Dan
Roth, Lauren
Rudolfsson, Gudrun
Ruffin, Mack
Ruiz, Marco
Ruotsalainen, Heidi
Russon, Lynne
Saavedra, JM
Sack, David A.
Salsbury, Stacie A
Sanada, Hiromi
Sanders, Kerrie
Scherag, Andre
Schmidt, Harold
Schmied, Virginia
Schoenberg, Nancy
Schulze, Kerry
Schwartz, Vicki S.
Schwartz, Marc D.
Scullin, Michael K.
Sekiguchi, Miho
Sexton, Claire E.
Sharma, Amrish
Shea, Judy A.
Sheerin, Neil S.
Shepherd, M. L.
Shneerson, Catherine
Shroba, Jodi
Silvestri, Nicholas J.
Slattery, David
Smart, Neil
Speck, Patricia M.
Sprigle, Stephen
Steene-Johannessen, Jostein
Stevens, Melissa B.
Stewart, Bob
Stock, Stephanie
Stolley, Melinda
Stovitz, Steven D.
Stricker, Linda J
Stulak, John M.
Swisher, Anne K.
Symonds, Michael E.
Szilagyi, Andrew
Tannen, Antje
Taylor, Anne
Tekle, M.
Thomas, Randal J.
Thompson, Juliana
Thompson, Kimberly M
Thraen, Iona
Timmerman, Kyle L.
Tonolo, Giancarlo
Tourigny, Jocelyne
Tzen, Yi-Ting
Unruh, Mark A.
Van Bodegraven, Ad A
Van Den Akker-van Marle, M.E.
Van Geijlswijk, Ingeborg M.
VanDenKerkhof, Elizabeth G
Verbrugghe, Mathieu
Verdú, José
Villari, Paolo
Vitetta, Luis
Wake, M.M.
Wanchai, Ausanee
Wang, Feng
Wang, Dongxu
Warren-Jeanpiere, Lari
Watts, Gerald
Weber, Griffin M.
Weeks, Lori E.
Weickert, Martin O.
Weiner, Shira Schecter
Weinstein, Ron
Whelehan, Patsy
White, Richard
White, Helen
Wilborn, Doris
Wilson, Leigh
Wingham, Jennifer
Winqvist, Ola
Wu, Yong-rui
Wu, Rebekah Ryanne
Xu, Fujie
Yager, Joel
Yang, Shiming
Yen, Chia-Hung
Yu, Ping
Yu, Margaret

